# Pathophysiology, diagnosis, and management of discogenic low back pain: a phenotype-driven precision framework for surgical and interventional decision-making

**DOI:** 10.3389/fsurg.2026.1787353

**Published:** 2026-05-25

**Authors:** Yanxu Feng, Yahao Li, Zhongqiu Sa, Zhilin Bai, Feng Mao, Jiangfeng Yu

**Affiliations:** 1 Department of Orthopedics, Kunshan Hospital of Integrated Traditional Chinese and Western Medicine, Kunshan/Suzhou, Jiangsu, China; 2 Nanjing University of Chinese Medicine, Nanjing, Jiangsu, China

**Keywords:** discogenic low back pain, myofascial trigger points, phenotype-based treatment, precision medicine, spine surgery, thoracolumbar fascia, ultrasound-guided dry needling

## Abstract

**Background:**

Discogenic Low Back Pain (DLBP) remains a major diagnostic and therapeutic challenge due to its heterogeneous pathophysiology and overlapping clinical presentation. Current management frequently relies on empirical stepwise strategies with limited mechanistic specificity. The distinct mechanisms of disc degeneration have drawn significant attention, highlighting the need for a phenotype-driven precision framework to support rational surgical and interventional decision-making.

**Methods:**

This narrative review evaluates literature from PubMed, Embase, and Web of Science up to April 2026, focusing on the pathophysiology and phenotype-based management of DLBP. Key terms included “discogenic low back pain,” “phenotype,” “basivertebral nerve ablation,” “thoracolumbar fascia,” “myofascial trigger point,” and “ultrasound-guided dry needling.” The review highlights the “triad” of disc degeneration—structural damage, functional impairment, and metabolic dysregulation—and integrates myofascial and thoracolumbar fascial dysfunction as functional modifiers that may coexist with structural disc phenotypes and influence diagnostic interpretation and therapeutic escalation.

**Results:**

Existing evidence supports the conceptual stratification of DLBP into four clinical phenotypes based on dominant pain-generating mechanisms. Vertebrogenic DLBP is characterized by endplate inflammation and Modic changes, for which basivertebral nerve (BVN) ablation is the primary supported intervention in appropriately selected patients. Annulogenic DLBP involves annular fissures associated with high-intensity zones (HIZ), where bipolar cooled radiofrequency ablation (biacuplasty) provides a targeted option. Mixed DLBP features concurrent endplate and annular pathology, potentially necessitating combined denervation strategies, whereas neuro-sensitized DLBP is dominated by central and peripheral sensitization, for which neuromodulation may serve as a salvage option. Across these phenotypes, functional myofascial or fascial involvement may be assessed using clinical examination and, when available, ultrasound-based dynamic evaluation. A staged therapeutic pathway places rehabilitation and manual therapy as early global strategies, ultrasound-guided dry needling as a potential intermediate functional intervention, and ablative, neuromodulatory, or surgical procedures as options for refractory or structurally dominant cases.

**Conclusion:**

DLBP management should move from generalized algorithms toward mechanism-informed precision care. Integrating structural imaging, functional soft-tissue assessment, and phenotype-specific interventions may improve individualized treatment selection while reducing unnecessary procedural escalation.

## Introduction

Low back pain (LBP) remains the leading cause of years lived with disability worldwide, imposing a substantial and growing socioeconomic burden. Among its multifactorial etiologies, intervertebral disc degeneration is widely recognized as a major contributor to chronic LBP. Discogenic Low Back Pain (DLBP), defined as pain originating from internal disc disruption in the absence of overt nerve root compression, is estimated to account for approximately 26%–42% of patients with chronic LBP (1). Establishing a definitive diagnosis of DLBP requires the rigorous differentiation of this condition from other primary axial pain generators, most notably facet-mediated and sacroiliac (SI) joint pain (2). DLBP is classically characterized by “loading intolerance,” wherein pain is predominantly midline and exacerbated by axial loads—such as prolonged sitting or forward flexion—and typically attenuated by recumbency. In clinical contrast, facet-mediated pain is characteristically provoked by spinal extension rather than flexion. Meanwhile, SI joint pain typically presents with localized tenderness over the posterior superior iliac spine and can be specifically reproduced through provocative maneuvers such as the FABER (Patrick's) test (3). Because clinical features alone often lack the pathognomonic specificity required for targeted interventional selection, a precise diagnosis necessitates the correlation of clinical symptoms with imaging biomarkers and, in refractory cases, confirmatory diagnostic blockades.

From a pathophysiological perspective, DLBP is characterized by progressive disruption of the annulus fibrosus microarchitecture, activation of inflammatory signaling pathways, and pathological ingrowth of blood vessels and nociceptive nerve fibers into normally aneural disc tissue (4). These biological changes establish a direct structural and biochemical substrate for pain generation. Although DLBP has traditionally been considered a degenerative condition of older adults, emerging epidemiological evidence suggests an increasing prevalence in younger populations, potentially associated with sedentary lifestyles and early mechanical overload. Degenerative disc changes relevant to DLBP have been reported with increasing frequency in individuals younger than 30 years, underscoring its growing clinical relevance (5).

In current clinical practice, management of DLBP typically follows a stepwise paradigm, progressing from conservative treatment to minimally invasive interventions and, ultimately, surgical reconstruction. While this approach is conceptually intuitive, its application in the intermediate stage of minimally invasive therapy remains highly heterogeneous. Uncertainty persists regarding appropriate indication criteria, patient selection, and the durability of clinical benefit across different interventional modalities. Importantly, these challenges are not solely attributable to technical limitations, but rather reflect the biological heterogeneity of disc degeneration and the presence of multiple, distinct pain-generating mechanisms that are not adequately addressed by uniform treatment algorithms. Accordingly, this review aims to synthesize recent advances in the understanding of DLBP pathophysiology and contemporary management strategies, with a particular focus on linking structural imaging biomarkers, functional myofascial/fascial assessment, and mechanistic phenotypes to staged therapeutic options. By integrating MRI-defined disc pathology with ultrasound-based dynamic soft-tissue evaluation and phenotype-specific interventions, this work seeks to provide a structured, mechanism-informed framework to support individualized clinical decision-making in patients with DLBP.

## Methods

PubMed, Embase, and Web of Science databases were searched for literature published up to April 2026. The primary keywords used included “discogenic low back pain”, “intervertebral disc degeneration”, “phenotype”, “basivertebral nerve ablation”, “biacuplasty”, “sinuvertebral nerve”, “spinal cord stimulation”, “total disc replacement”, “thoracolumbar fascia”, “myofascial trigger points”, “manual therapy”, and “ultrasound-guided dry needling”. Additional relevant studies were identified through manual screening of reference lists from key review articles and consensus statements.

The inclusion criteria were: (1) clinical studies (including randomized controlled trials and cohort studies) and high-quality reviews evaluating interventions for discogenic low back pain; (2) pivotal basic science or translational studies focusing on the pathophysiology of disc degeneration; and (3) articles published in English. The exclusion criteria were: (1) studies predominantly focusing on radicular pain or other compressive spinal pathologies; (2) non-English publications; and (3) conference abstracts or unpublished data. High-quality literature was selected, reviewed, and summarized to construct the proposed phenotype-driven precision framework.

## Pathophysiological basis of DLBP

### Core pathomechanics and pain mechanisms

**Figure 1:**
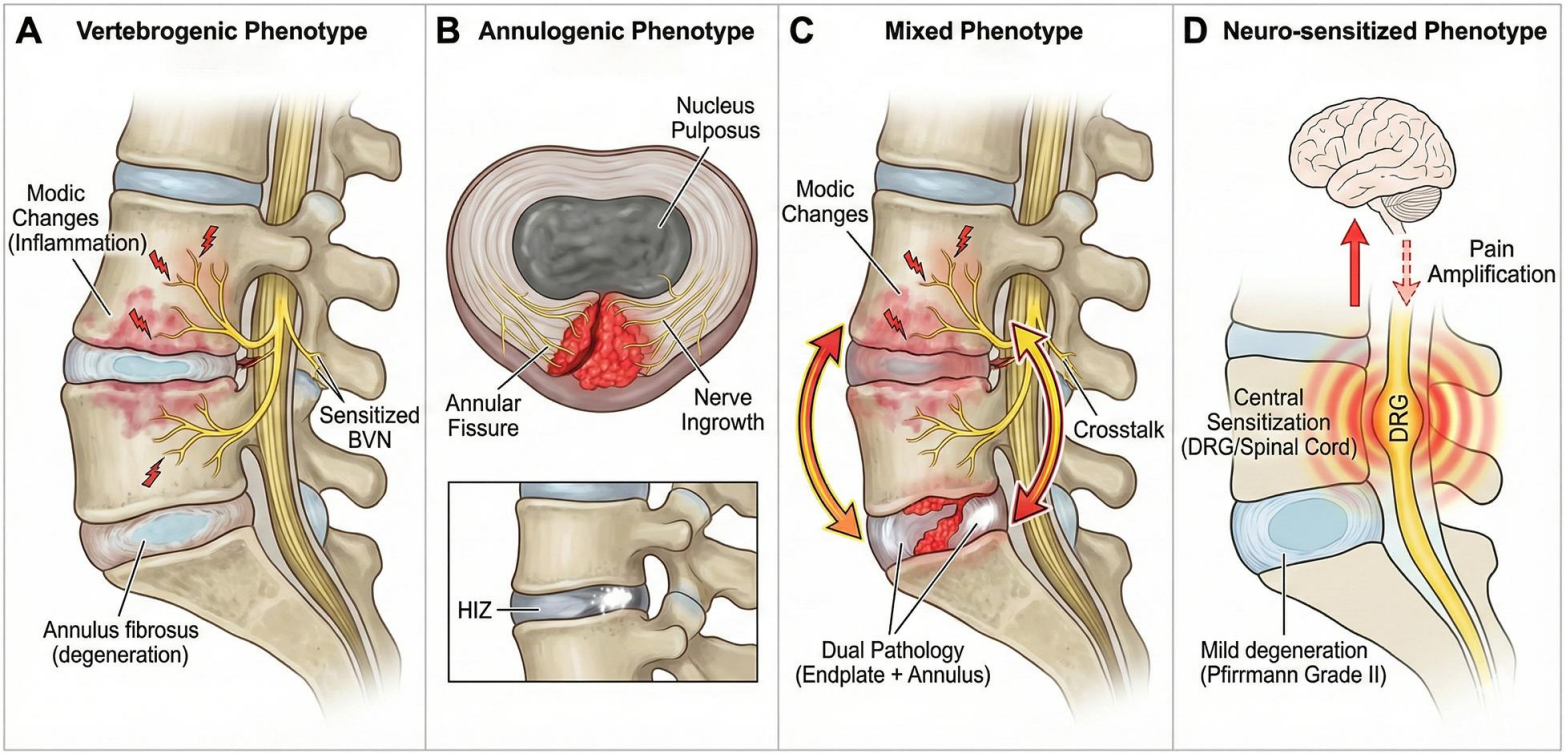
Integrated pathophysiological mechanisms of discogenic low back pain (DLBP). **(A)** The classical triad of disc degeneration includes structural damage, functional impairment, and metabolic dysregulation. **(B)** Degenerative annular and endplate pathology may promote inflammatory signaling, neovascularization, nociceptive nerve ingrowth, and peripheral sensitization. **(C)** Persistent nociceptive input may induce spinal and supraspinal sensitization, resulting in pain amplification. **(D)** Myofascial and thoracolumbar fascial dysfunction may act as cross-cutting functional modifiers by increasing peripheral nociceptive input and contributing to symptom heterogeneity across structural phenotypes. IL-1β, interleukin-1 beta; TNF-α, tumor necrosis factor-alpha; VEGF, vascular endothelial growth factor; NGF, nerve growth factor; MTrPs, myofascial trigger points; TLF, thoracolumbar fascia.

DLBP arises from a dynamic interaction between three interrelated pathological domains: structural damage, functional impairment, and metabolic dysregulation (Figure 1). Together, these mechanisms contribute to the complex pain-generating substrates within the degenerating intervertebral disc. Although theoretically distinct in early pathological stages, these substrates frequently overlap in clinical practice, making precise clinical isolation challenging (1, 6). Nevertheless, this multi-domain interplay provides the biological basis for the marked heterogeneity observed in clinical presentation and treatment response.

#### Structural damage

Structural degeneration of the intervertebral disc is initiated by fissuring of the annulus fibrosus and progressive dehydration of the nucleus pulposus, ultimately leading to disc height loss and segmental instability. These changes are commonly visualized on magnetic resonance imaging (MRI) as hypointense (“black”) discs. As demonstrated by Feng et al. (7), disruption of normal disc architecture compromises the disc's capacity to distribute axial loads, resulting in abnormal stress transmission to the adjacent vertebral endplates. Importantly, such structural failure establishes a permissive environment for subsequent biomechanical overload and biochemical signaling cascades.

#### Functional impairment

Beyond gross structural alterations, disc degeneration is associated with profound changes in segmental biomechanics. Degenerated discs exhibit impaired load-sharing capability, leading to focal stress concentration at the vertebral endplates and adjacent motion segments. Biomechanical studies conducted in both *in vivo* and *in vitro* settings indicate that the lumbosacral junction (L4–S1) is particularly vulnerable to fatigue-induced mechanical derangement (8). Under repetitive loading conditions, these segments demonstrate increased compressive strain accompanied by diminished shear-strain dissipation—a “high-compression, low-shear” mechanical phenotype. This abnormal loading pattern amplifies stress at the endplate–nucleus interface and may precipitate endplate microfailure, thereby accelerating degenerative progression and pain generation.

#### Metabolic dysregulation

Structural disruption of the annulus fibrosus or vertebral endplate exposes the normally immune-privileged nucleus pulposus to the systemic immune environment, triggering a sustained inflammatory response. This process is characterized by upregulation of proinflammatory cytokines, including interleukin-1β (IL-1β) and tumor necrosis factor-α (TNF-α), which disrupt extracellular matrix homeostasis and promote catabolic degeneration. In parallel, these mediators stimulate neurotrophic and angiogenic signaling pathways, facilitating pathological ingrowth of blood vessels and sensory nerve fibers—most notably sinuvertebral nerve (SVN) and dorsal root ganglion–derived afferents—into deeper layers of the disc. This neuroimmune interaction represents a central biological driver of discogenic pain (9, 10).

### Integrated pathophysiological cascade underlying DLBP

The progression from disc degeneration to clinically overt DLBP reflects a multistep neurovascular and neuroimmune cascade involving nerve ingrowth, peripheral sensitization, and central sensitization.

#### Nerve ingrowth

Under physiological conditions, the intervertebral disc is largely aneural and avascular, with innervation restricted to the outer annulus fibrosus. Degenerative matrix disruption, in conjunction with cytokine upregulation (notably IL-1β and TNF-α), induces local expression of neurotrophic and angiogenic factors such as nerve growth factor (NGF), brain-derived neurotrophic factor (BDNF), and vascular endothelial growth factor (VEGF). This altered microenvironment permits pathological penetration of C-fiber nociceptors and microvessels into the inner annulus and nucleus pulposus—an anatomical prerequisite for discogenic pain (11, 12). Experimental work by Binch et al. demonstrated that sensory nerve sprouting closely parallels inflammatory cytokine expression during disc degeneration, reinforcing the mechanistic link between inflammation and nociception (13).

#### Peripheral sensitization

Following aberrant nerve infiltration, chronic exposure to a proinflammatory milieu—comprising IL-1β, TNF-α, prostaglandin E2 (PGE2), and chemokines—alters nociceptor excitability (14). These mediators lower activation thresholds and amplify responsiveness to mechanical stimuli at both peripheral terminals and the dorsal root ganglion. Clinically, peripheral sensitization manifests as allodynia and hyperalgesia, providing a biological explanation for posture-dependent pain and exaggerated mechanical sensitivity commonly observed in patients with symptomatic DLBP (15).

#### Central sensitization

Sustained nociceptive input from sensitized peripheral afferents may induce hyperexcitability within spinal dorsal horn neurons, resulting in long-term potentiation of central pain pathways. This process, termed central sensitization, is characterized by expanded receptive fields and amplification of pain perception that may persist even after attenuation of the initiating peripheral stimulus. Mechanistic synthesis by Mohd Isa et al. highlights the role of glutamatergic/NMDA receptor signaling and neuroimmune crosstalk in establishing durable “pain memory,” thereby contributing to chronicity and treatment resistance in a subset of DLBP patients (6).

#### The biomechanical-neurobiological-fascial Continuum

Beyond the classical triad of disc degeneration, chronic DLBP should be conceptualized within an expanded biomechanical-neurobiological-fascial continuum. Discogenic instability and altered load distribution may induce compensatory hypertonicity within the paraspinal musculature and thoracolumbar fascia (TLF). This sustained myofascial overactivity may promote localized ischemia, facilitate the development of myofascial trigger points, and impair fascial gliding, thereby contributing to peripheral nociceptive input (16–19). Importantly, degenerating intervertebral discs and paraspinal muscles are coupled in a self-reinforcing pathological loop. Progressive intervertebral disc degeneration (IVDD) is associated with increased fatty infiltration of paraspinal muscles, which correlates with both structural disease severity and pain intensity (20, 21). In turn, muscle dysfunction further exacerbates segmental instability and abnormal load transmission, thereby accelerating disc degeneration and functional decline. These persistent myofascial abnormalities may serve as continuous sources of peripheral nociceptive input, capable of initiating and maintaining central sensitization (16, 17). Accordingly, structural disc pathology and muscle-fascia dysfunction should not be regarded as independent entities, but rather as interdependent components of an integrated pain-generating system, particularly in mixed and neuro-sensitized phenotypes (18, 19).

Within the proposed phenotype-driven framework, myofascial and fascial dysfunction should therefore be interpreted not as a separate competing diagnosis, but as a functional modifier superimposed on structural or neurobiological DLBP phenotypes. For example, vertebrogenic or annulogenic pain may initiate compensatory paraspinal guarding, whereas persistent TLF stiffness and MTrPs may amplify peripheral nociceptive input and promote neuro-sensitization. This modifier concept may help explain why patients with comparable MRI-defined disc pathology exhibit markedly different pain intensity, disability, and treatment responses. Therefore, phenotype assignment should incorporate both structural pain generators and dynamic functional contributors (16–19, 22–25).

### Phenotype-based treatment stratification

**Figure 2:**
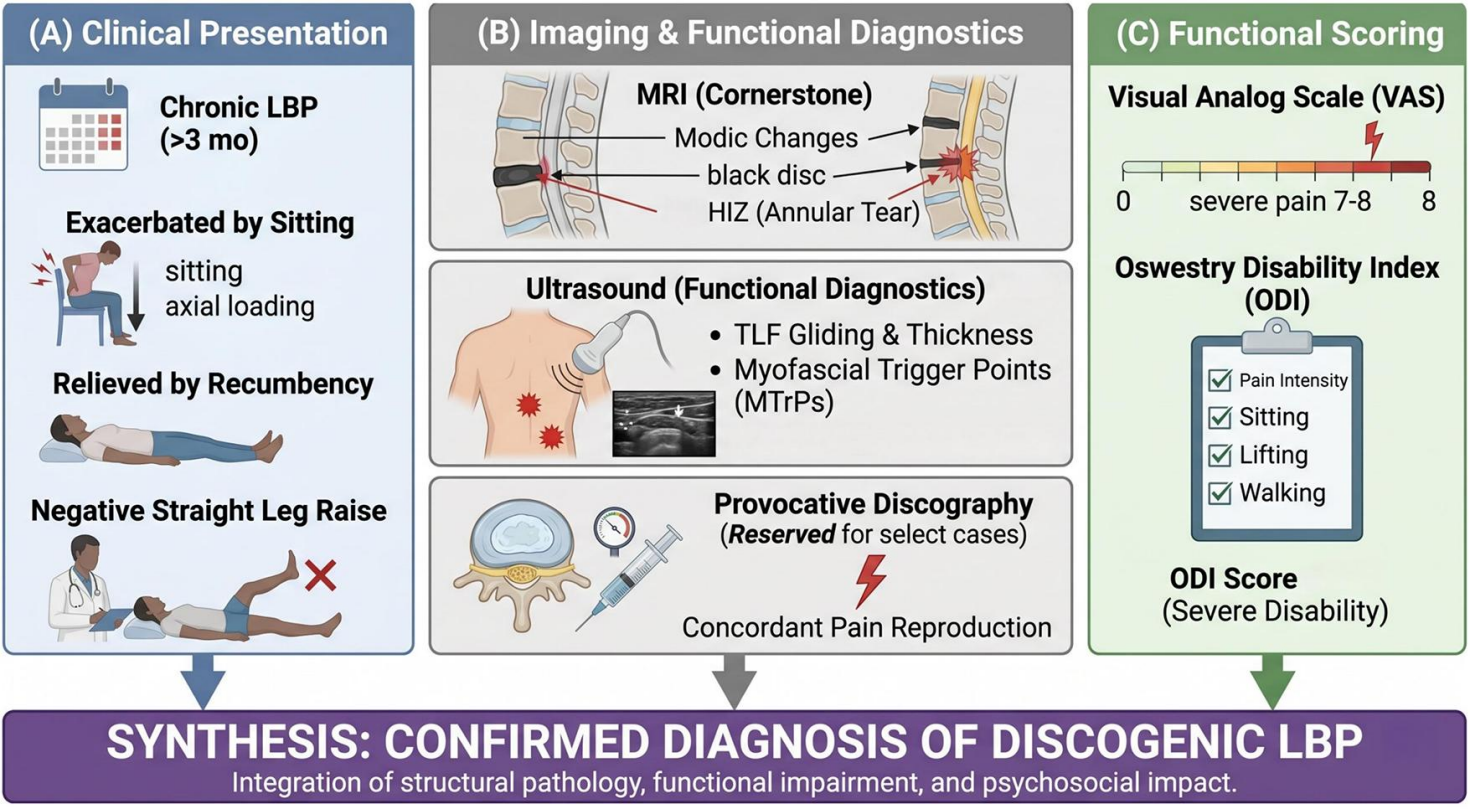
Phenotype-based stratification of DLBP with functional myofascial/fascial modifiers. Four dominant phenotypes are proposed: **(A)** vertebrogenic DLBP, characterized by Modic changes and basivertebral nerve (BVN) sensitization; **(B)** annulogenic DLBP, characterized by annular fissures or high-intensity zones (HIZs); **(C)** mixed DLBP, characterized by coexisting endplate and annular pathology; and **(D)** neuro-sensitized DLBP, characterized by pain disproportionate to structural findings and features of peripheral or central sensitization. Across these phenotypes, MTrPs, paraspinal hypertonicity, TLF stiffness, and impaired fascial gliding may function as symptom-amplifying modifiers rather than independent competing phenotypes.

To facilitate the transition from empirical, symptom-based care toward precision-oriented management, we propose a clinically pragmatic classification framework that stratifies DLBP according to four dominant structural or neurobiological pain-generating mechanisms, while explicitly incorporating myofascial and thoracolumbar fascial dysfunction as a cross-cutting functional modifier (16–19). In this model, vertebrogenic, annulogenic, mixed, and neuro-sensitized phenotypes are not defined solely by MRI abnormalities, but by the concordance among clinical presentation, structural imaging biomarkers, functional soft-tissue findings, and response to diagnostic or therapeutic trials. This phenotype-based approach aligns dominant pain generators with staged therapeutic strategies and serves as a decision-support model rather than a prescriptive treatment guideline (Figure 2) (16–19, 22–26). The clinical comparison of the four DLBP phenotypes, including dominant mechanisms, core biomarkers, functional modifiers, and staged interventions, is summarized in Table 1.

**Table 1:** Clinical comparison of DLBP phenotypes, diagnostics, and interventions.

Phenotype	Dominant mechanism	Core biomarkers	Functional modifier/assessment	Staged intervention
Vertebrogenic	Endplate inflammation and BVN sensitization.	Modic type 1 or 2 changes.	Paraspinal guarding or TLF stiffness as secondary amplifiers; evaluate clinically and with ultrasound when symptoms are disproportionate.	Rehabilitation/manual therapy plus functional care when needed; BVN ablation for clearly concordant vertebrogenic pain.
Annulogenic	Annular fissures with nociceptive ingrowth.	Posterior HIZ or annular fissure.	Segmental spasm, deep MTrPs, or impaired fascial mobility may magnify mechanically provoked pain.	Early rehabilitation/manual therapy, US-DN for persistent functional pain, then biacuplasty for concordant annular pain.
Mixed	Concurrent endplate and annular pathology.	Overlapping Modic changes and HIZs.	Functional modifiers are often clinically relevant because dual pathology may sustain compensatory paraspinal overactivity.	Assess and treat reversible functional pain generators before combined BVN/SVN denervation.
Neuro-sensitized	Dominant peripheral and central sensitization.	Pain disproportionate to structural MRI findings; CSI/QST abnormalities.	MTrPs and fascial dysfunction may maintain peripheral input, but palpation specificity may be reduced by widespread hyperalgesia.	Multidisciplinary care and reversible functional interventions before neuromodulation in refractory cases.

#### Vertebrogenic DLBP

This phenotype is primarily driven by vertebral endplate inflammation, typically manifested as Modic type 1 or 2 changes on MRI, and associated sensitization of the intraosseous basivertebral nerve (BVN) (27). Clinically, pain is characteristically deep, midline, and exacerbated by axial loading. Diagnosis relies on the identification of Modic changes on MRI, often supplemented by confirmatory diagnostic blocks targeting the BVN. Among available minimally invasive interventions, BVN ablation represents the most extensively studied modality for this phenotype, with multiple Level I randomized controlled trials demonstrating sustained clinical benefit over conservative management at 2–5 years in appropriately selected patients (28, 29). Coexisting paraspinal muscle guarding or TLF stiffness may be interpreted as a functional amplifier rather than an isolated primary structural target (16–21). When present, these findings support concomitant rehabilitation, manual therapy, or ultrasound-guided functional interventions and should be considered complementary to BVN-targeted therapy in patients with clearly concordant vertebrogenic pain (30–33).

#### Annulogenic DLBP

Annulogenic pain arises from nociceptive ingrowth into radial or circumferential annular fissures, commonly visualized as high-intensity zones (HIZs) on T2-weighted MRI (34). For patients with isolated annular pathology, bipolar cooled radiofrequency ablation (biacuplasty) has emerged as a targeted interventional option that mechanistically delivers controlled thermal energy directed at ablating these infiltrating nociceptive nerve endings. In contrast to earlier thermal-based techniques such as intradiscal electrothermal therapy (IDET), biacuplasty has demonstrated clinically meaningful improvements in pain and function in sham-controlled randomized trials involving carefully selected patients (35). Because annular pain is commonly mechanically provoked, it may induce segmental protective spasm and localized MTrPs (16, 17). Functional soft-tissue assessment may help determine whether early manual therapy or targeted US-DN is appropriate before considering intradiscal thermal procedures, particularly when symptoms are disproportionate to annular imaging findings (16, 17, 22–25, 32, 33).

#### Mixed DLBP

Mixed DLBP represents a substantial subset of refractory cases in which both vertebral endplate dysfunction and annular disruption coexist. In this context, single-modality interventions frequently yield suboptimal outcomes due to incomplete coverage of concurrent pain generators. Emerging evidence suggests that combined denervation strategies—such as concurrent targeting of the BVN and SVN—may enhance responder rates compared with single-target approaches, as reported in recent prospective studies (36). This phenotype underscores the importance of comprehensive mechanistic assessment prior to intervention. Mixed DLBP may be a phenotype in which functional myofascial and fascial modifiers are particularly clinically relevant, because concurrent endplate and annular pathology can produce persistent compensatory paraspinal overactivity. Therefore, treatment planning should avoid direct escalation to combined denervation until modifiable myofascial pain generators have been assessed and, where appropriate, addressed (16–25).

#### Neuro-sensitizedDLBP

Neuro-sensitized DLBP is characterized by disproportionate pain severity relative to structural imaging findings and reflects a dominant contribution from peripheral and central sensitization mechanisms. To clinically distinguish this phenotype from purely annulogenic pain—which typically presents with mechanically reproducible, localized symptoms—neuro-sensitized patients exhibit widespread allodynia, expanded receptive fields, and often a lack of response to localized diagnostic nerve blocks. In this framework, we advocate the use of objective assessment tools, such as the Central Sensitization Inventory (CSI) or quantitative sensory testing (QST), to accurately identify this phenotype (37, 38). Patients often exhibit features such as hyperalgesia, pain chronicity, and psychological comorbidities. In this setting, ablative interventions directed at local disc structures are frequently ineffective. Management therefore prioritizes neuromodulation strategies. Recent comparative data suggest that dorsal root ganglion stimulation (DRG-S) may provide improved focal pain control compared with conventional spinal cord stimulation (SCS) in selected patients with refractory discogenic pain (39). In neuro-sensitized DLBP, MTrPs and fascial dysfunction may serve as persistent peripheral inputs maintaining central sensitization. However, these findings should be interpreted cautiously, because widespread hyperalgesia may reduce the specificity of palpation-based diagnosis. Ultrasound-based assessment may therefore be useful for identifying objective soft-tissue abnormalities and for selecting reversible, function-restoring interventions before considering neuromodulation (16–19, 22–25, 37–39).

### Diagnostic criteria

**Figure 3:**
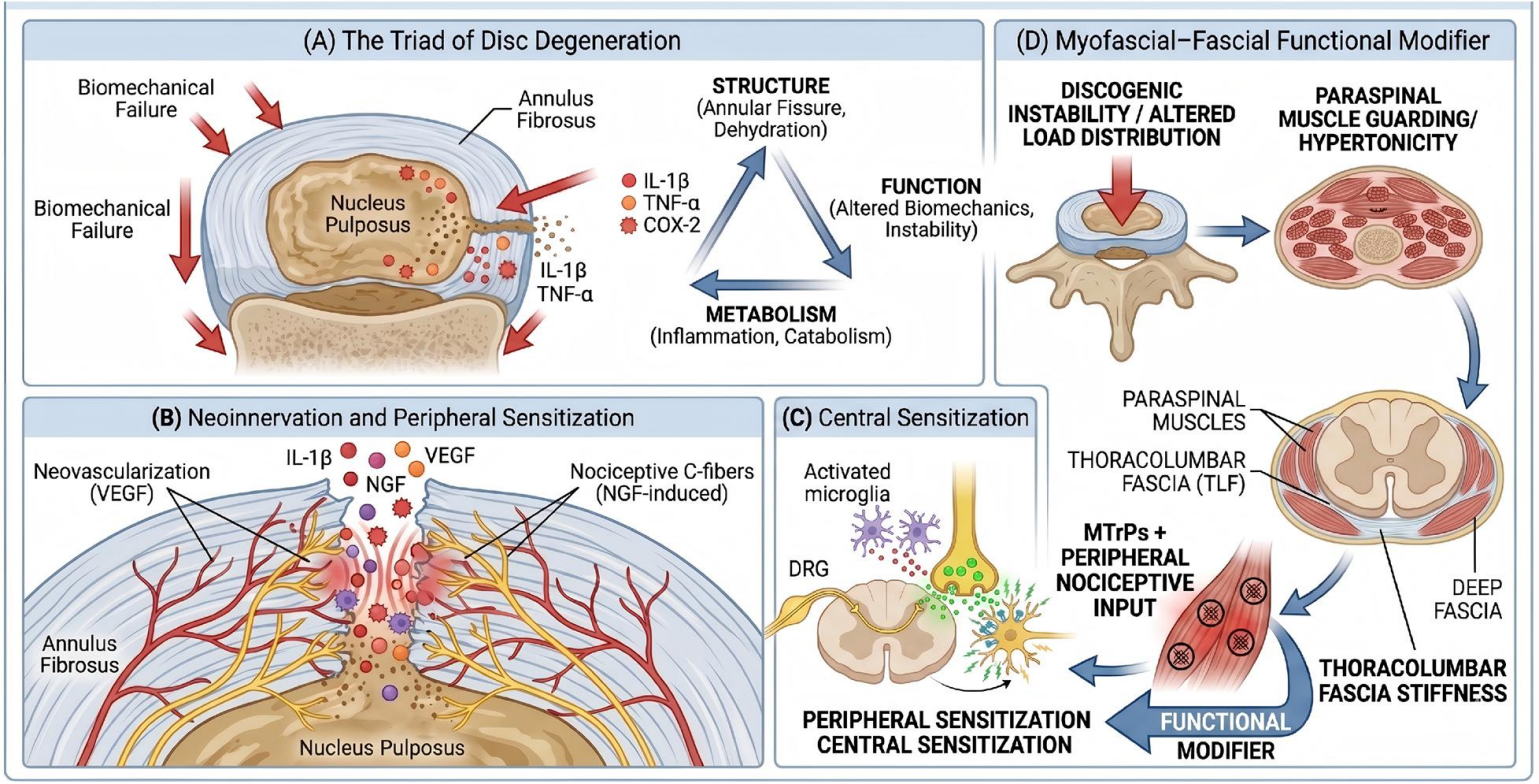
Integrated diagnostic framework for DLBP. (**A**) The triad of disc degeneration includes structural disruption, functional impairment/instability, and metabolic inflammation or catabolism. (**B**) Neoinnervation and peripheral sensitization may occur through inflammatory mediator release, neovascularization, and nociceptive C-fiber ingrowth into the annulus fibrosus. (**C**) Persistent peripheral nociceptive input may contribute to dorsal root ganglion activation, microglial activation, and central sensitization. (**D**) Myofascial-fascial functional modifiers, including paraspinal muscle guarding, myofascial trigger points, and thoracolumbar fascia stiffness, may amplify or perpetuate discogenic pain. Diagnosis requires synthesis of clinical presentation, MRI-based structural phenotyping, ultrasound-based functional assessment, and validated functional or sensitization measures. MRI evaluates Modic changes, disc signal loss, and HIZs, whereas ultrasound may dynamically assess TLF morphology, fascial gliding, shear strain, and MTrPs when functional modifiers are suspected. Patient-reported outcome measures, the Central Sensitization Inventory (CSI), and quantitative sensory testing (QST) help quantify pain severity, disability, and sensitization. Provocative discography should be reserved for selected surgical candidates when non-invasive assessments remain inconclusive.

Accurate diagnosis of DLBP requires rigorous integration of clinical presentation with imaging biomarkers and, in selected cases, invasive diagnostic testing (Figure 3). No single modality is sufficient in isolation; rather, concordance across multiple domains is highly valuable for reliable phenotype assignment and appropriate treatment selection (40).

#### Clinical features

Patients typically present with midline lumbar pain persisting for more than three months, exacerbated by axial loading (e.g., prolonged sitting or forward flexion) and relieved by recumbency. This pattern of “loading intolerance” may help differentiate discogenic pain from facet-mediated pain, which is often aggravated by extension. However, as highlighted by Peng et al. (1), clinical features alone lack diagnostic specificity and primarily function as a screening tool to identify candidates for advanced imaging.

#### Imaging

MRI remains the cornerstone of non-invasive assessment, although its interpretation requires careful contextualization. A recent prospective study demonstrated that deep learning–accelerated abbreviated MRI protocols maintain diagnostic performance comparable to conventional imaging for degenerative lumbar pathologies, including disc degeneration and Modic changes, supporting MRI as a first-line diagnostic modality (41). ① The “Black Disc”, defined by loss of T2-weighted signal intensity, reflects disc dehydration and proteoglycan depletion. While this finding reliably indicates degeneration, it is not pathognomonic for pain, as similar changes are frequently observed in asymptomatic individuals (7). ② HIZs within the posterior annulus are widely regarded as markers of painful annular disruption. Histopathological correlation suggests that HIZs represent vascularized granulation tissue within annular fissures; nevertheless, their positive predictive value remains limited without concordant clinical symptoms (4). ③ Modic Changes, particularly types 1 and 2, are increasingly recognized as imaging biomarkers of a vertebrogenic pain phenotype. These changes correlate strongly with basivertebral nerve sensitization and may guide phenotype-specific interventional strategies (27).

#### Ultrasound-based functional diagnostics

While MRI remains the established reference standard for structural phenotyping, it inherently cannot capture real-time soft-tissue dysfunction. High-resolution musculoskeletal ultrasound may usefully bridge this gap, enabling dynamic, real-time assessment of TLF morphology—including thickness, echogenicity, deformation, shear strain, and gliding properties—as well as sonographic visualization of myofascial trigger points (42). Recent systematic analyses have confirmed that advanced ultrasound imaging shows promise as a reliable, non-invasive tool for characterizing TLF pathology and dysfunction and for monitoring treatment responses (22). Contemporary data suggest that patients with chronic low back pain exhibit increases in TLF thickness and reduced fascial gliding compared with asymptomatic individuals (23). In LBP populations, deep fascia and associated soft tissues generally demonstrate greater thickness than in controls (24). This functional imaging modality may be particularly valuable for DLBP, in whom structural MRI findings alone often correlate poorly with symptom burden (25). While such patterns remain exploratory at this stage, accumulating evidence supports the integration of ultrasound-based functional diagnostics into a comprehensive, multimodal assessment algorithm for DLBP (22).

In the proposed decision-making algorithm, ultrasound-based functional diagnostics may complement structural MRI and clinical screening, particularly when pain severity is disproportionate to MRI findings, deep paraspinal MTrPs are suspected on palpation, or impaired fascial mobility is clinically suspected. By identifying potentially reversible functional pain generators, ultrasound may help select patients who are more likely to benefit from rehabilitation, manual therapy, or US-DN (22–25). This approach may reduce premature escalation to ablative denervation or reconstructive procedures in patients whose symptoms are substantially driven by functional soft-tissue modifiers.

#### Provocative discography

Historically considered the reference standard for linking disc morphology to symptoms, provocative discography remains controversial. The primary diagnostic benefit lies in confirming the specific symptomatic level prior to surgical reconstruction, but this must be explicitly weighed against substantial procedural risks. High false-positive rates have been reported, especially in patients with central sensitization or psychological overlay, and evidence suggests that disc puncture may accelerate degeneration (43, 44). However, this risk appears to be needle-size dependent. Intradiscal injections and provocative testing can be performed safely with minimal structural disruption when the needle thickness is appropriately proportional to the disc height (45). Despite this mechanical safety optimization, the routine use of discography is strongly discouraged. Specifically, it is contraindicated in patients with the neuro-sensitized phenotype or significant psychological overlay due to a high propensity for false-positive responses (45), and should not be performed if the findings will not fundamentally alter the established treatment plan (46). Current consensus recommends reserving discography for select cases in which surgical intervention is being contemplated and non-invasive assessments remain inconclusive (47). When employed, strict diagnostic criteria are imperative, including prior exclusion of alternative pain generators via diagnostic blocks, concordant pain reproduction at low intradiscal pressures, and negative control levels to minimize false-positive responses (48).

#### Functional quantification

Patient-reported outcome measures (PROMs) are essential for capturing the multidimensional impact of DLBP. The Visual Analog Scale (VAS) and Oswestry Disability Index (ODI) are widely used to quantify pain severity and functional limitation, respectively. Beyond descriptive assessment, these instruments enable calculation of the minimal clinically important difference (MCID), allowing differentiation between statistically significant changes and clinically meaningful improvement, as demonstrated in recent high-quality trials (49).

### Therapeutic strategies: a step-wise approach

The step-wise therapeutic framework proposed here is not strictly based on a rigid chronological progression (i.e., simply advancing to the next tier after a set time period), but is fundamentally mechanism-driven. Escalation between steps is determined by the dominant pain-generating phenotype, structural disease stage, and failure to achieve clinically meaningful improvement (Minimal Clinically Important Difference, MCID) after an adequate trial of the preceding level of care.

For practical implementation, the therapeutic hierarchy can be conceptualized as four clinically relevant tiers. The first tier consists of education, pharmacological modulation, active rehabilitation, and manual therapy, aiming to restore global biomechanical control and reduce neuromuscular overactivity. The second tier addresses persistent functional pain generators through ultrasound-based assessment and targeted myofascial interventions, such as US-DN, when localized MTrPs or fascial dysfunction are identified. The third tier consists of phenotype-specific minimally invasive structural interventions, including BVN ablation for vertebrogenic pain, biacuplasty for annulogenic pain, and combined BVN/SVN strategies for selected mixed phenotypes. The final tier is surgical reconstruction, reserved for refractory, structurally dominant disease with severe disability and strict clinico-radiological concordance after failure of conservative, functional, and targeted interventional care. The key clinical evidence supporting phenotype-based interventional strategies is summarized in Table 2.

**Table 2:** Key evidence supporting phenotype-based interventional strategies.

Phenotype/Intervention	Author	Level of evidence	Study Design	Sample Size	Follow-up	Key Clinical Outcomes
	(Year)			(n)		
Vertebrogenic (BVN Ablation)	McCormick et al. (2024) (60)	Level II	Pooled Analysis (3 Prospective Studies)	247 (1-yr)	1-5 Years	↓ opioid use (−61.7%), ↓ injections (−76.4%); fusion: 6.5%
				205 (5-yr)		
	McCormick et al. (2025) (3)	Level IV	Prospective Cohort (Real-World)	60	12 Months	NRS −2.8; ODI −14.0; ≥50% pain relief: 49.1%; fusion: 1.9%
Annulogenic (Biacuplasty)	Desai et al. (2016) (35)	Level I	Prospective RCT	63	6 Months	VAS -2.4 vs CMM -0.56; 50% vs 18% responders; superior to CMM
	Elawady et al. (2024) (70)	Level I	Prospective RCT	44	6 Months	Superior to conservative care; VAS 7.0→2.0; ODI 45.8→23.3
Mixed Phenotype (Combined)	Moneim et al. (2024) (65)	Level I	Meta-Analysis	429	12 Months	ODI −28.1; VAS −3.16 vs controls
Neuro-sensitized(Neuromodulation)	Mons et al. (2023) (77)	Level IV	Prospective Study	15	12 Months	NRS 7.2→4.3; ODI 41.2→25.8; 42.5% pain relief
	Mons et al. (2024) (39)	Level II	Prospective Comparative Study	29	12 Months	Both Burst and DRGS ↓ NRS/ODI; DRGS significantly lower NRS at 12 mo
	Kallewaard et al. (2019) (82)	Level IV	Prospective Cohort	20	12 Months	NPRS 7.20→2.29; ODI 42.1→20.1
	Kirketeig et al. (2023) (78)	Level III	Retrospective Registry	411	1–10 Years	48% had ≥30% pain relief at 1 yr; 21% explantation at 10 yr

Level of evidence is classified based on standard orthopedic hierarchical criteria: Level I (High-quality RCTs or Meta-analyses); Level II (Prospective comparative cohort studies); Level III (Retrospective cohort studies or Case-control studies); Level IV (Case series, including single-arm prospective cohorts without a control group).conventional medical management (CMM).

#### Conservative management: the first line

**Figure 4:**
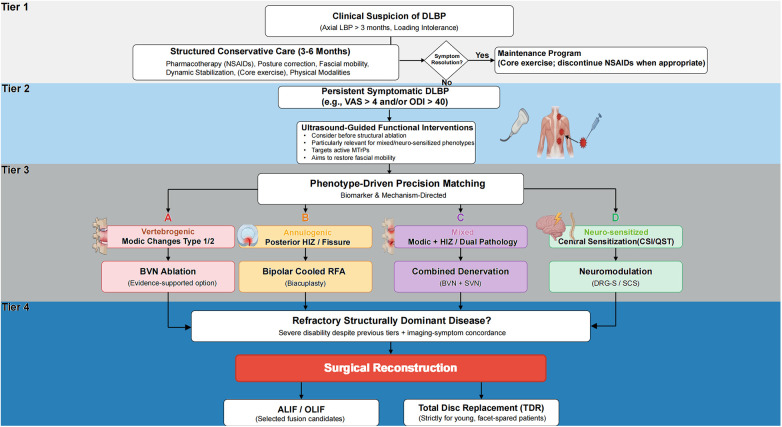
Staged phenotype-based treatment algorithm for DLBP. Tier 1 emphasizes education, pharmacological modulation, active rehabilitation, posture correction, fascial mobility restoration, core stabilization, and manual therapy. Tier 2 addresses persistent functional modifiers using ultrasound-based assessment and targeted functional interventions such as ultrasound-guided dry needling (US-DN) when concordant MTrPs, paraspinal hypertonicity, or impaired TLF gliding are identified. Tier 3 applies phenotype-specific interventions, including BVN ablation for vertebrogenic pain, biacuplasty for annulogenic pain, combined BVN/sinuvertebral nerve strategies for selected mixed cases, and neuromodulation for refractory neuro-sensitized cases. Tier 4 is reserved for surgical reconstruction, including anterior/oblique lumbar interbody fusion (ALIF/OLIF) or total disc replacement (TDR), in refractory structurally dominant cases after multidisciplinary reassessment.

For patients presenting with uncomplicated DLBP at the initial phase of clinical management (i.e., newly diagnosed, without severe structural instability or progressive neurologic deficits), a structured conservative regimen of 3 to 6 months is widely recommended as the initial therapeutic approach. This phase is not merely “waiting” but active rehabilitation aimed at mitigating the pro-inflammatory milieu and restoring segmental stability (50) (Figure 4).

#### Pharmacological modulation (targeting the inflammatory cascade)

Nonsteroidal anti-inflammatory drugs (NSAIDs): Selective COX-2 inhibitors (e.g., celecoxib) are the first-line therapeutic agents. Bedaiwi et al. demonstrated their superior efficacy over acetaminophen, attributing this to their ability to directly inhibit prostaglandin synthesis within the annular fissures (51). Upon symptom resolution, systemic NSAIDs should be discontinued to avoid long-term risks, and maintenance should rely on core exercises.

Neuropathic Agents: While frequently prescribed, gabapentinoids show limited efficacy in isolated DLBP lacking a radicular component. Evidence suggests their use should be restricted to cases with confirmed central sensitization (52).

Opioid Stewardship: In alignment with 2022 CDC guidelines, opioid administration should be limited to acute exacerbations (<7 days). Their utility in chronic discogenic pain is negligible and outweighed by the risk of dependency and opioid-induced hyperalgesia (53).

#### Biomechanical rehabilitation (restoring dynamic stability)

Physical therapy may be optimally shifted from passive modalities to active, function-oriented rehabilitation during the early- and intermediate-care phases. Essential components include targeted posture correction to optimize load distribution, specific stretching protocols to restore fascial mobility, and dynamic core stabilization to mitigate secondary myofascial overload (54, 55). The primary biomechanical objective involves recruiting the transversus abdominis and lumbar multifidus muscles to function as a “natural corset,” thereby offloading axial stress from the compromised intervertebral disc. Emerging high-quality evidence supports the integration of yoga-based interventions into the conservative management of discogenic low back pain. In a recent randomized controlled trial, Sahu et al. (56) reported that a 12-week yoga protocol yielded statistically significant reductions in VAS and ODI scores. These benefits may be attributable to dual mechanisms that concurrently improve spinal flexibility and downregulate sympathetic nervous system tone, thereby addressing the biomechanical and psychosocial dimensions of chronic pain. Collectively, this evidence underscores that active, function-oriented rehabilitation—incorporating posture correction, fascial-mobility restoration, core stabilization, and adjunctive mind-body practices such as yoga—constitutes an essential initial tier within the phenotype-driven precision framework for discogenic low back pain.

Manual therapy may be incorporated within this early-stage conservative tier as a global biomechanical and neuromuscular modulatory strategy (30, 31). Its primary role is to improve spinal mobility, reduce protective muscle guarding, enhance fascial mobility, and facilitate participation in active rehabilitation. However, manual therapy is inherently limited by its dependence on surface palpation and its reduced precision for deeper segmental paraspinal structures. Therefore, persistent localized deep MTrPs or sonographically identifiable fascial dysfunction after adequate manual therapy may support consideration of targeted ultrasound-guided functional interventions before structural denervation.

#### Physical modalities

Traction, spinal manipulation, and other manual or physical modalities may provide short-term symptom modulation by reducing muscle guarding, improving spinal mobility, and transiently altering mechanical loading (30, 31, 57). However, their long-term disease-modifying effects on disc degeneration remain limited. Therefore, these approaches should be viewed as adjunctive strategies that facilitate active rehabilitation rather than stand-alone definitive treatments.

Failure of conservative management is defined as persistent pain and functional limitation despite a minimum of 3–6 months of structured therapy, without attainment of established MCID thresholds in validated PROMs.

#### Ultrasound-guided myofascial and fascial treatments

When symptoms persist despite early-stage rehabilitation and manual therapy, targeted ultrasound-guided functional interventions may serve as an intermediate therapeutic tier before escalation to ablative denervation or surgical reconstruction. This tier is particularly relevant when clinical examination and ultrasound identify persistent MTrPs, paraspinal muscle hypertonicity, or impaired TLF gliding that is concordant with the patient's pain pattern.

Addressing modifiable functional pain generators may therefore represent an important intermediate step within the phenotype-driven treatment hierarchy for DLBP (58). In this context, US-DN has been proposed as a minimally invasive technique for deactivating MTrPs and improving fascial mobility, with preliminary evidence suggesting potential clinical utility in low back pain associated with myofascial trigger points (32, 33, 42). By employing real-time high-resolution ultrasound, practitioners may achieve more precise localisation of affected musculature and fascia compared with conventional palpation-guided techniques, potentially reducing the risk of injury to adjacent neurovascular structures. This real-time visual control substantially improves procedural accuracy, increases the likelihood of eliciting a local twitch response, and reduces both the number of required treatment sessions and the number of needled MTrPs per session compared with blind needling (32).

Beyond its role in identifying MTrPs within paraspinal muscles and the TLF, ultrasound guidance also facilitates dynamic assessment of fascial gliding, thickness, and shear strain-parameters that have been suggested as correlates of pain and functional impairment in chronic low back pain populations, although the clinical significance of these findings remains to be established (22, 23, 59). It has been hypothesised that US-DN may exert its therapeutic effect in part through modulation of peripheral nociceptive input arising from compensatory muscle spasm, thereby potentially interrupting a proposed vicious cycle of myofascial overactivity, local ischemia, and central sensitisation (32, 33). This mechanistic consideration suggests that US-DN could be particularly applicable to mixed and neuro-sensitized DLBP phenotypes, in whom structural MRI findings alone often correlate poorly with symptom severity. However, the current evidence base remains limited; most studies are small, non-randomised, or lack sham controls, and high-quality randomised controlled trials are needed to confirm these preliminary observations.

Given its minimally invasive nature and the absence of permanent structural alterations, US-DN may be reasonably positioned as a reversible, function-restoring intervention prior to escalation toward more invasive ablative or surgical therapies, provided that patients are appropriately counselled about the uncertainty of therapeutic benefit. Its integration into a comprehensive, multimodal assessment algorithm for DLBP is conceptually aligned with the broader principles of predictive, preventive, and personalised medicine (PPPM), yet this remains a proposal requiring prospective validation rather than an established standard of care (18). Thus, US-DN is not proposed as a replacement for BVN ablation, biacuplasty, neuromodulation, or surgery, but as a complementary functional intervention that may reduce peripheral nociceptive input and improve patient selection for subsequent structural procedures.

### Minimally invasive interventions

Structural minimally invasive interventions constitute the phenotype-specific structural tier of the treatment hierarchy, rather than the default next step after conservative care. These modalities are indicated for patients with persistent pain and a clearly defined pathological phenotype following 3–6 months of structured conservative care and, when relevant, after assessment or treatment of modifiable myofascial/fascial contributors (26). Therapeutic decision-making should be tailored to the dominant pain generator, structural disease stage, functional soft-tissue modifiers, and patient comorbidities, prioritising reversible and lower-risk strategies whenever clinically appropriate (27).

#### Nerve ablation

##### Basivertebral nerve (BVN) ablation

###### Mechanism of action

This technique utilises a transpedicular approach under fluoroscopic guidance to target and ablate the BVN within the vertebral body. It aims to interrupt nociceptive signals mediated by endplate micro-damage and inflammation. The procedure is specifically indicated for “vertebrogenic low back pain,” radiographically characterised by Modic Type 1 or 2 changes on MRI (27).

###### Clinical efficacy

Evidence has accumulated progressively from controlled trials to real-world settings. McCormick et al. (60) first conducted a pooled analysis of three prospective clinical trials focusing on healthcare utilization. They demonstrated that opioid utilization decreased by 61.7% and the demand for spinal injections dropped by 76.4% over a mean 5-year follow-up period. The rate of conversion to spinal fusion was only 6.5%, highlighting significant resource-saving value. Subsequently, the same group validated the clinical efficacy in a prospective, real-world cohort study (*n* = 60). Results showed a mean Numeric Rating Scale (NRS) reduction of 2.8 and an ODI decrease of 14.0 points at 1 year, with 49.1% of patients achieving ≥50% pain relief. Notably, the fusion rate remained low at 1.9% in this real-world setting (61). This stepwise validation from strict trial populations to general clinical practice reinforces the role of BVN ablation as a stable, fusion-sparing alternative for vertebrogenic LBP.

###### Safety profile

The safety profile is excellent, with a low incidence of adverse events, primarily limited to transient lower limb paraesthesia or puncture site pain. Serious neurological injuries or infections are rarely reported in the current literature (62).

###### Clinical recommendation

BVN ablation is a well-supported minimally invasive intervention specifically for patients with the Vertebrogenic phenotype. However, its widespread adoption warrants critical appraisal. The procedure's efficacy is highly contingent upon rigid MRI selection criteria (specifically Modic type 1 or 2 changes), which introduces inherent selection bias and limits its applicability for the broader DLBP population. Furthermore, the high upfront cost of specialized radiofrequency equipment and the current lack of multi-decade comparative data against conservative care or fusion surgery necessitate careful cost-effectiveness considerations in routine clinical practice.

##### Sinuvertebral nerve (SVN) ablation

###### Mechanism of Action

Radiofrequency ablation (RFA) of the SVN disrupts nociceptive fibres transmitting pain from annular injury and degeneration. It is frequently employed in conjunction with BVN ablation to address “mixed” pain phenotypes and enhance overall analgesic coverage (63).

###### Clinical Efficacy

While RCTs strictly isolating SVN ablation are limited, emerging case series suggest short-term efficacy. In a retrospective series of 12 patients with residual DLBP following fusion, SVN RFA resulted in a significant reduction in median VAS from 7.00 (IQR 6.00–7.75) preoperatively to 1.00 (IQR 1.00–1.00) during follow-up (*p* = 0.002) (64). Furthermore, a recent systematic review and meta-analysis synthesising SVN and BVN data indicated that RFA provided significant improvements in ODI (mean difference −28.08) and VAS (mean difference −3.16) compared to controls (65). However, the inherent physiological potential for peripheral nerve regeneration raises valid controversies regarding the definitive long-term durability of isolated SVN ablation (66, 67).

###### Safety Profile

Due to the anatomical proximity to the nerve root and dural sac, there is a risk of transient radicular irritation or paraesthesia. Current small-scale reports suggest this incidence is low and self-limiting. Long-term complication data remains to be established.

###### Clinical Recommendations

SVN ablation is not recommended as a monotherapy but rather as an adjunct to BVN ablation or other neuromodulatory procedures (65). It is particularly applicable for patients with Mixed DLBP involving both endplate and annular pathology. Precision imaging guidance is highly recommended to avoid collateral damage to neural and vascular structures. Given the current low level of evidence and the potential for nerve regeneration, thorough patient counselling regarding limitations is highly advised (68).

#### Intradiscal thermal procedures

##### Bipolar cooled radiofrequency ablation (biacuplasty)

###### Mechanism of Action

Cooled radiofrequency probes are introduced via a bilateral posterior trans-annular approach to ablate nociceptive nerve endings at 50–55 °C. This controlled low-temperature profile minimises structural damage to the collagen matrix, thereby avoiding further iatrogenic destabilization of the disc.

###### Clinical efficacy

A prospective, randomized multicenter trial by Desai et al. (69) evaluated biacuplasty in DLBP patients diagnosed via provocation discography. The authors reported that the biacuplasty cohort achieved a mean VAS score reduction of 2.4 and a 50% treatment responder rate at 6 months post-operatively, alongside improvements in functional measures such as the ODI. These outcomes were superior to conventional medical management (which showed a mean VAS reduction of 0.56 and an 18% responder rate), supporting the utility of biacuplasty as a therapeutic intervention. Furthermore, a recent controlled study by Elawady et al. (70) validated its performance in a real-world setting; the authors reported that the Biacuplasty cohort achieved a profound reduction in VAS scores from a baseline of 7.0 to 2.0 and ODI scores from 45.8 to 23.3 at 6 months post-operatively. These outcomes were significantly superior to conservative management (which showed modest improvements to VAS 4.5 and ODI 34.4), further supporting the utility of biacuplasty as an intermediate therapeutic intervention.

###### Safety profile

While the procedure is generally well-tolerated with a low incidence of catastrophic events, it inherently carries the standard risks associated with percutaneous intradiscal access, including potential discitis, transient radicular pain, and injection-site morbidity. Furthermore, because the procedure involves delivering concentrated thermal energy to the annulus, theoretical concerns regarding accelerated long-term disc degeneration or biomechanical destabilization remain, necessitating prolonged radiographic follow-up to definitively confirm its structural safety profile (26).

###### Clinical recommendations

For patients with isolated annular pathology (Annulogenic DLBP), biacuplasty has emerged as a targeted interventional option that mechanistically delivers controlled thermal energy to ablate the infiltrating nociceptive nerve endings associated with annular fissures. While it has demonstrated clinically meaningful improvements in pain and function in DLBP patients, a critical appraisal reveals inherent limitations. Biacuplasty acts primarily as a palliative neuroablative measure rather than a structural disease-modifying treatment. Although it denervates the painful tissue, it lacks the capacity for direct structural repair of the disrupted collagen matrix. Because the physical fissure typically persists, the underlying biomechanical instability of the intervertebral disc may not be fully addressed, a factor well-documented to drive progressive structural degeneration over time (71). Consequently, clinicians should consider that this intervention primarily manages pain symptoms without directly reversing the underlying structural pathology.

##### Intradiscal electrothermal therapy (IDET)

###### Mechanism of action

A thermal resistive catheter delivers heat (up to 90 °C) to the posterior annulus, aiming to induce collagen coagulation, nociceptor ablation, and potential fissure closure (72).

###### Clinical efficacy

Evidence for IDET is historical and heterogeneous. While Pauza et al. (73) reported statistically significant but clinically modest improvements (VAS ↓1.3, ODI ↓7) at 6 months, other studies failed to demonstrate superiority over sham (74). Due to inconsistent efficacy and strict selection dependency, IDET has largely been superseded by biacuplasty, which offers more reproducible outcomes and a superior safety margin (75).

###### Safety profile

The high-temperature application carries a risk of thermal injury to the vertebral endplates and adjacent neural structures. Reports of accelerated disc degeneration and height loss have raised concerns regarding its biomechanical safety (72).

###### Clinical recommendations

Given the availability of safer and more targeted alternatives, IDET is rarely considered a mainstream clinical option today. Its historical application relied heavily on invasive provocative discography—a diagnostic prerequisite that has been linked to an increased risk of accelerated iatrogenic disc degeneration. Consequently, the routine clinical use of IDET is generally no longer recommended. The trajectory of this technology primarily highlights the structural limitations of early unipolar intradiscal thermal therapies and underscores the necessity of rigorous, sham-controlled negative trials in evaluating interventional efficacies (76).

#### Neuromodulation

##### Spinal cord stimulation (SCS)

###### Mechanism of action

Modern SCS modalities (e.g., BurstDR™, 10-kHz) modulate central pain processing without generating paraesthesia, making them suitable for axial back pain refractory to prior interventions (77).

###### Clinical efficacy

In a prospective study, Mons et al. (77) demonstrated the targeted efficacy of SCS in 17 patients with severe, treatment-refractory DLBP. Following a successful trial phase in 15 patients, permanent SCS implantation yielded significant improvements; at 12 months, mean ODI scores decreased from 41.2 to 25.8, and NRS pain scores dropped from 7.2 to 4.3. Complementing these clinical findings, a broader retrospective registry study by Kirketeig et al. (78) evaluated the real-world trajectory of SCS. Their analysis revealed that while 48% of patients maintained a ≥30% pain reduction at 1 year, the 10-year explantation risk reached 21%. Notably, higher educational attainment and active employment were strong predictors of therapeutic success, whereas advanced age (≥60 years) and 10 kHz stimulation waveforms significantly increased explantation risk. Together, these studies highlight that while SCS provides robust relief for refractory DLBP, its long-term sustainability in real-world settings is critically mediated by socioeconomic and demographic factors.

###### Safety profile

While the risk of permanent neurological deficit is inherently low, device-related complications are not insignificant. Hardware-related issues, such as lead migration, lead fracture, or surgical site infection, remain notable clinical challenges that can necessitate costly revision surgeries and mitigate initial therapeutic benefits (79).

###### Clinical recommendations

SCS may be considered for patients with refractory DLBP dominated by the neuro-sensitized phenotype, typically after the exhaustion of structural minimally invasive options. A trial stimulation (10–14 days) is conventionally required, with permanent implantation reserved for patients achieving ≥50% pain relief (80). However, a critical appraisal highlights substantial clinical and economic limitations. The high upfront capital cost of the neuromodulation system, coupled with the potential need for future hardware revisions, imposes a significant economic burden, making its overall cost-effectiveness a subject of ongoing debate (79). Furthermore, clinical outcomes in neuromodulation can be notably influenced by psychological factors and placebo responses. Consequently, rigorous multidisciplinary screening—including comprehensive psychological evaluation—is strongly advised to mitigate selection bias and minimize the risk of long-term therapy failure (81).

##### Dorsal root ganglion stimulation (DRG-S)

###### Mechanism of action

DRG-S targets the L2 dorsal root ganglion—a critical gatekeeper for lumbar disc afferents—to modulate neuronal excitability and block nociceptive transmission.

###### Clinical efficacy

Emerging prospective studies suggest targeted efficacy for focal anatomical pain. L2-DRG stimulation has shown profound pain reduction (∼60%–70%) and functional improvement at 12 months (82). Comparative analyses suggest DRG-S may offer advantages over Burst SCS in NRS and ODI reduction for specific discogenic cohorts (39).

###### Safety profile

Complication rates are reported between 8% and 12% (primarily lead migration/infection), with good short-term safety (39, 83).

###### Clinical recommendations

Considered a “salvage therapy” for refractory cases of the neuro-sensitized phenotype, particularly those unresponsive to or intolerant of SCS. It requires precise patient selection and operator expertise. Furthermore, neuromodulation—particularly DRG-S—is not a straightforward procedural technique; it involves a steep learning curve and requires specialized multidisciplinary training for both physicians and allied clinical staff (e.g., specialized nurses) to optimize trial programming, patient education, and long-term device management (84, 85).

#### Intradiscal injections

##### Methylene blue injection

###### Mechanism of action

Acts via inhibition of nitric oxide synthase, sodium channel blockade, and chemical neurolysis to provide anti-inflammatory and analgesic effects for annular tear-related pain (86).

###### Clinical efficacy

Efficacy is controversial. A meta-analysis by Deng et al. (87) found statistically significant short-term (3-month) pain reduction, but benefits were not sustained at 6 months compared to placebo.

###### Safety profile

High concentrations are cytotoxic to nucleus pulposus and annulus fibrosus cells, posing a risk of accelerated degeneration (88).

###### Clinical recommendations

Not recommended for routine use in any specific phenotype. May be considered as a temporary analgesic measure in acute exacerbations or for patients refusing other interventions, provided dosage is limited (88).

##### Regenerative biologics (PRP/MSC)

###### Mechanism of action

Platelet-Rich Plasma (PRP) and Mesenchymal Stem Cells (MSCs) aim to modulate inflammation and promote matrix repair via growth factor release and paracrine signalling (89, 90).

###### Clinical efficacy

In a recent prospective randomized controlled trial, Wang et al. (91) demonstrated that intradiscal PRP injection yielded superior long-term outcomes compared to steroid injections for DLBP. At the 1-year follow-up, the PRP cohort exhibited significantly lower pain scores (VAS: 2.11 vs. 3.48) and reduced functional disability (ODI: 14.59 vs. 23.36) relative to controls. Furthermore, MRI evaluations revealed a significant improvement in Pfirrmann grades within the PRP group, suggesting active disc remodeling and structural restoration. Complementing this, a comprehensive systematic review of clinical studies by Vadalà et al. (92) evaluated the efficacy of MSC therapies for DLBP. Their analysis confirmed that MSC interventions provide significant and sustained pain relief and functional improvement for up to 1 to 3 years. While the safety profile is excellent with no reported serious adverse events or tumorigenesis, the review noted that definitive structural restoration on MRI remains variable across patients, emphasizing their continued investigational status. Beyond intradiscal applications, ultrasound-guided PRP injections have also shown potential benefit for adjacent paraspinal muscle injuries and fascial tears. In a randomized comparative study of 30 professional athletes with acute muscle trauma, Bubnov et al. reported that ultrasound-guided PRP injection combined with conservative treatment significantly accelerated pain relief (from day 1 onward), expedited recovery of strength and range of motion (at 7 and 14 days), and shortened the mean time from injury to return to sport (10 ± 1.2 days vs. 22 ± 1.5 days with conservative treatment alone), suggesting that a comprehensive soft-tissue regenerative strategy may extend beyond the disc itself (93).

###### Safety profile

Short- to mid-term safety is acceptable, but long-term data (>5 years) regarding risks such as heterotopic ossification are lacking.

###### Clinical recommendations

Currently considered investigational and not yet integrated into the phenotype-driven framework. These therapies are not recommended as routine clinical practice and are more appropriately restricted to clinical trials or as a last resort before surgery (92).

A phenotype-driven treatment algorithm holds significant promise for optimizing the modern management of DLBP, but its clinical value depends on avoiding a purely procedure-centered interpretation. Early management should emphasize education, pharmacological modulation, active rehabilitation, and manual therapy to restore global biomechanical and neuromuscular control. When persistent functional pain generators, such as MTrPs or thoracolumbar fascial dysfunction, are identified, ultrasound-guided interventions such as US-DN may serve as an intermediate and reversible treatment option. Structural minimally invasive procedures should then be selected according to the dominant phenotype, including BVN ablation for vertebrogenic pain, biacuplasty for annulogenic pain, and combined BVN/SVN strategies for carefully selected mixed cases. Neuromodulation and surgery should be reserved for refractory cases after multidisciplinary reassessment. This staged approach may facilitate the transition from empirical treatment to mechanism-based care while reducing unnecessary procedural escalation.

### The final tier: surgical reconstruction

Surgical intervention is generally reserved for a highly selected cohort experiencing incapacitating, refractory discogenic pain (e.g., ODI > 60) that persists despite a minimum of 6 months of comprehensive conservative care and targeted minimally invasive management. Given the irreversible nature of spinal arthrodesis or arthroplasty, mitigating the risk of surgical failure requires a rigorous, multidisciplinary patient selection process. First, candidates must demonstrate definitive single-level (or maximum two-level) structural pathology with absolute clinico-radiological concordance. This mandates that morphological aberrations identified on MRI precisely correlate with the patient's mechanical symptom profile, and that alternative pain generators have been systematically excluded via diagnostic blocks (47). Furthermore, comprehensive biopsychosocial screening is imperative. Patients whose clinical presentation is driven predominantly by central sensitization (identifiable via tools such as the CSI, maladaptive neuroplasticity, or severe untreated psychiatric comorbidities) must be explicitly excluded from surgical consideration (94). Operating on neuro-sensitized phenotypes inevitably leads to high rates of Failed Back Surgery Syndrome (FBSS) and exacerbated functional disability, as structural reconstruction cannot resolve centrally mediated pain states (95). In correctly phenotyped patients, the surgical goal shifts definitively from symptom “modulation” to “anatomical reconstruction,” aiming to eliminate the nociceptive motion segment while optimizing segmental kinematics and sagittal balance.

#### Lumbar fusion

Anterior Lumbar Interbody Fusion (ALIF) and Oblique Lumbar Interbody Fusion (OLIF) are generally favored over posterior approaches for pure discogenic pain to spare paraspinal musculature (96). The anterior approach allows for comprehensive discectomy (removing the anterior annular nociceptive source), placement of a larger footprint graft (reducing the risk of subsidence), and superior restoration of segmental lordosis compared to posterior techniques. Scott-Young et al. (96) recently reported on a large cohort of discogenic pain patients treated with stand-alone ALIF. The study demonstrated an overall fusion rate of 99.6% and “Excellent/Good” clinical outcomes in >85% of patients, with significant improvements in VAS and ODI sustained at 2 years. However, clinicians must acknowledge procedure-specific complication profiles, as anterior approaches carry unique risks of mortality, deep venous thrombosis, and gastrointestinal events (97), while long-term surgical failure can manifest as pseudoarthrosis (non-union), cage subsidence, or adjacent segment disease (ASD) requiring subsequent revision (96). Consequently, for patients without significant central canal stenosis requiring direct decompression, anterior-based fusion is increasingly advocated as the preferred surgical strategy to maximize indirect decompression and sagittal alignment.

#### Total disc replacement (TDR)

For younger patients with preserved facet joint function and absence of significant spinal instability, TDR offers a motion-preserving alternative to fusion. Unlike fusion, TDR aims to restore physiological kinematics, theoretically reducing the risk of ASD. The debate regarding long-term superiority remains active. A recent 2024 Meta-analysis comparing TDR vs. Fusion indicated that while TDR offers a slight advantage in early postoperative recovery and range of motion, long-term (>5 years) pain relief and functional outcomes are statistically comparable between the two cohorts (98).Despite avoiding pseudoarthrosis, TDR introduces unique device-specific complication and failure modes, including implant migration, subsidence, bearing wear, heterotopic ossification, and the delayed onset of facet joint arthrosis over time (99).While technical demands are higher and indication criteria are stricter (contraindicated in severe facet arthrosis), long-term studies suggest TDR can provide comparable pain relief to fusion with superior functional preservation in correctly selected DLBP candidates (100, 101).

Surgical reconstruction represents the definitive tier for incapacitating DLBP (ODI > 60) refractory to minimally invasive care. For pure discogenic pathology, ALIF is often favored over posterior approaches due to its superior capacity for radical discectomy and lordotic restoration (102). The debate regarding the long-term superiority of TDR vs. fusion remains highly active. While TDR was biomechanically designed to restore physiological kinematics and theoretically mitigate ASD, clinical evidence suggests its long-term advantages are not absolute. Systematic reviews of mid- to long-term results (mean follow-up up to 17.3 years) (103) indicate that while TDR demonstrated significant improvements in VAS and ODI scores at final follow-up, there is insufficient evidence to conclude that TDR is superior to fusion surgery. Although TDR provides early kinematic benefits and high patient satisfaction rates (75.5–93.3%), the clinical trajectories for pain relief and functional improvement ultimately converge with those of fusion over time. Furthermore, radiographic evidence reveals that while TDR preserves segmental motion, it reduces but does not completely eliminate the incidence of long-term ASD, with reported postoperative adjacent segment degeneration occurring in 2.2% to 17% of cases depending on the study cohort and device success. Crucially, TDR success is highly dependent on selecting young candidates with pristine facet joints. Thus, the surgical decision must be personalised, dictated by the patient's facet joint integrity and sagittal balance parameters.

### Emerging regenerative therapies

Current therapeutic paradigms are exploring a gradual transition from palliative or ablative approaches toward regenerative strategies aimed at restoring disc structure and biological function. However, it is imperative to clearly distinguish between early clinical feasibility data and preclinical investigations to avoid overinterpreting their current clinical utility.

#### Hydrogels

The clinical evaluation of hydrogel augmentation predominantly remains in the early feasibility phase. Injectable hydrogel scaffolds are designed to reconstitute the biomechanics of the nucleus pulposus. In a clinical evaluation of a novel hydrogel scaffold, Beall et al. (104) reported a robust therapeutic response, documenting a reduction in mean NRS pain scores from 7.0 to 1.0. While promising, hydrogels primarily address the mechanical component of DLBP. Their long-term success relies on the integrity of the annulus fibrosus to prevent material extrusion. Therefore, they are best indicated for patients with “contained” degeneration (Pfirrmann Grade III-IV) rather than those with severe annular incompetence.

#### Gene therapy

Evidence supporting these molecular interventions is largely derived from preclinical models. Novel delivery systems utilizing viral vectors or exosomes to transport transcription factors, such as FOXF1, have demonstrated the capacity to reprogram degenerated disc cells toward a healthy phenotype in preclinical models. Tang et al. (105) suggest these molecular interventions represent a potential class of disease-modifying therapies for intervertebral disc degeneration. Specifically, their *in vivo* study demonstrated that engineered extracellular vesicles delivering the FOXF1 transcription factor significantly restored disc height, tissue hydration, and proteoglycan content, while concurrently mitigating pain behaviors in a murine model. It is critical to emphasize that these molecular interventions remain confined to *in vivo* animal testing phases. While they conceptually represent a potential class of disease-modifying therapies, directly extrapolating outcomes from murine models to the complex biomechanical and neuroimmune environment of the human spine is premature. Significant translational hurdles must be overcome before human clinical applications can be rigorously evaluated.

## Discussion

The management of DLBP is evolving toward an integrative, phenotype-based precision medicine approach, moving beyond a generic, structurally isolated strategy. Accumulating evidence increasingly supports this transition. This evolution aligns seamlessly with the broader concept of PPPM. By incorporating multimodal diagnostics (MRI for structural phenotyping, ultrasound for dynamic myofascial assessment) and applying targeted therapies across the biomechanical-neurobiological-fascial continuum, clinicians can construct an integrative treatment framework (Figure 4). The foundation of this approach relies on diagnostic precision and targeted conservative care. When non-operative measures fail, escalation is ideally guided by the dominant pathophysiological mechanism rather than a rigid procedural ladder. For instance, vertebrogenic pain driven by endplate inflammation has demonstrated favorable responses to BVN ablation, whereas annulogenic pain may be addressed by intradiscal modalities like biacuplasty. For patients with advanced structural failure or intractable symptoms, judicious surgical reconstruction (e.g., ALIF or TDR) remains a definitive therapeutic option.

A key implication of this integrative model is that structural imaging phenotypes should not be interpreted in isolation. Myofascial and fascial dysfunction may act as clinically relevant modifiers that amplify symptoms, perpetuate peripheral nociceptive input, and contribute to central sensitization. Incorporating these functional modifiers into the algorithm may improve patient selection by identifying individuals who are more likely to benefit from rehabilitation, manual therapy, or targeted ultrasound-guided functional interventions before structural denervation or reconstruction is pursued.

While this phenotype-driven framework offers a structured and mechanistic approach to DLBP management, its translation into real-world clinical practice presents several inherent challenges. Foremost is the complexity of overlapping pathologies. Purely isolated structural phenotypes are relatively uncommon; patients frequently present with concurrent annulogenic fissures (e.g., HIZs) and endplate-driven nociception (e.g., Modic changes). This anatomical overlap significantly complicates decision-making, as applying a highly targeted ablation may fail to address secondary pain generators, thereby compromising overall therapeutic efficacy.

Furthermore, the inherent limitations of MRI diagnostic specificity present a substantial hurdle. It is well-documented that morphological aberrations are frequently observed in asymptomatic populations, often representing incidental stages of natural aging rather than definitive nociceptive sources. Consequently, relying exclusively on isolated MRI findings carries a significant risk of false-positive diagnoses and phenotypic misattribution. Therefore, the identification of a structural phenotype on MRI strongly requires comprehensive clinical correlation and the systematic exclusion of alternative pain generators (e.g., via diagnostic medial branch blocks) before committing to any targeted intervention.

Beyond structural diagnostics, relying exclusively on an MRI-driven model may fail to capture the functional and biopsychosocial dimensions of chronic pain. DLBP is frequently complicated by MTrPs, TLF stiffness, altered neuromuscular control, central sensitization, neuroplasticity, and psychosocial comorbidities. Applying this framework without functional and multidisciplinary evaluation risks treating radiographic abnormalities in isolation rather than the patient's broader pain-generating system. Therefore, the proposed model should be interpreted as an integrated clinical framework in which MRI-defined structural phenotypes are combined with ultrasound-based functional assessment, PROMs, sensory testing, and patient-centered evaluation.

Moving forward, the therapeutic objective in DLBP is shifting from symptomatic palliation toward structural and biological restoration. Emerging disease-modifying therapies, including injectable hydrogels and exosome-based gene therapies, hold promise for intervening earlier in the degenerative process and potentially reversing elements of the “triad of degeneration.” However, it must be acknowledged that the algorithmic model proposed herein is a conceptual synthesis derived from retrospective data and existing pathophysiological evidence. Large-scale, prospective randomized controlled trials are needed to determine whether treatment stratification based on integrated structural and functional phenotypes can improve long-term outcomes compared with conventional empirical management.

## Conclusion

Progress in DLBP management is unlikely to arise from a single therapeutic modality, but rather from the systematic application of a phenotype-specific and mechanism-driven treatment algorithm. Importantly, precision care should not be limited to MRI-defined disc pathology. Functional myofascial and thoracolumbar fascial contributors should be considered in diagnosis and treatment selection, particularly when symptoms are disproportionate to structural imaging findings. A staged approach—beginning with rehabilitation and manual therapy, progressing to targeted ultrasound-guided functional interventions when appropriate, and reserving ablative, neuromodulatory, or surgical procedures for refractory and structurally dominant cases—may optimize current interventions while minimizing unnecessary procedural escalation.
